# Genetic Overlap Between Midfrontal Theta Signals and Attention-Deficit/Hyperactivity Disorder and Autism Spectrum Disorder in a Longitudinal Twin Cohort

**DOI:** 10.1016/j.biopsych.2023.05.006

**Published:** 2023-11-15

**Authors:** Ümit Aydin, Máté Gyurkovics, Cedric Ginestet, Simone Capp, Corina U. Greven, Jason Palmer, Gráinne McLoughlin

**Affiliations:** a Social, Genetic & Developmental Psychiatry Centre, Institute of Psychiatry, Psychology & Neuroscience, King’s College London, London, United Kingdom; b School of Psychology & Clinical Language Sciences, University of Reading, Reading, United Kingdom; c Centre for Cognitive Neuroscience, Institute of Neuroscience and Psychology, University of Glasgow, Glasgow, United Kingdom; d Bioinformatics and Health Statistics, Institute of Psychiatry, Psychology & Neuroscience, King’s College London, London, United Kingdom; e Radboud University Medical Center, Donders Institute for Brain, Cognition and Behaviour, Department of Cognitive Neuroscience, Nijmegen, the Netherlands; f Karakter Child and Adolescent Psychiatry University Centre, Nijmegen, the Netherlands; g School of Mathematical and Data Sciences, West Virginia University, Morgantown, West Virginia

**Keywords:** ADHD, ASD, EEG, Frontal midline theta, Longitudinal, Twin study

## Abstract

**Background:**

Cognitive control has been strongly linked to midfrontal theta (4–8 Hz) brain activity. Such control processes are known to be impaired in individuals with psychiatric conditions and neurodevelopmental diagnoses, including attention-deficit/hyperactivity disorder (ADHD) and autism spectrum disorder (ASD). Temporal variability in theta, in particular, has been associated with ADHD, with shared genetic variance underlying the relationship. Here, we investigated the phenotypic and genetic relationships between theta phase variability, theta-related signals (the N2, error-related negativity, and error positivity), reaction time, and ADHD and ASD longitudinally in a large twin study of young adults to investigate the stability of the genetic relationships between these measures over time.

**Methods:**

Genetic multivariate liability threshold models were run on a longitudinal sample of 566 participants (283 twin pairs). Characteristics of ADHD and ASD were measured in childhood and young adulthood, while an electroencephalogram was recorded in young adulthood during an arrow flanker task.

**Results:**

Cross-trial theta phase variability in adulthood showed large positive phenotypic and genetic relationships with reaction time variability and both childhood and adult ADHD characteristics. Error positivity amplitude was negatively related phenotypically and genetically to ADHD and ASD at both time points.

**Conclusions:**

We showed significant genetic associations between variability in theta signaling and ADHD. A novel finding from the current study is that these relationships were stable across time, indicating a core dysregulation of the temporal coordination of control processes in ADHD that persists in individuals with childhood symptoms. Error processing, indexed by the error positivity, was altered in both ADHD and ASD, with a strong genetic contribution.

SEE COMMENTARY ON PAGE 767

Goal-directed action in everyday life is dependent on cognitive control, a collection of cognitive and behavioral processes that are involved in prioritizing task-relevant representations and suppressing task-irrelevant representations (1,2). Cognitive control is known to be affected in a number of psychopathologies (3, 4, 5, 6, 7), including attention-deficit/hyperactivity disorder (ADHD) and autism (8, 9, 10, 11, 12, 13, 14, 15).

To better understand cognitive control, studies have investigated its neural correlates (7,15, 16, 17). Electrophysiological signals of interest include event-related potentials (ERPs), such as the error-related negativity (ERN), the error positivity (Pe), and the N2 component. The ERN occurs within 100 ms of an incorrect response, most prominently in tasks that require the inhibition of competing response tendencies (e.g., conflict tasks) or the suppression of a prepotent response tendency (e.g., Go/NoGo tasks) and thus has been considered a signal of automatic error monitoring (18,19). The Pe, in turn, is a positive deflection that follows the ERN on incorrect trials, occurring about 150 to 300 ms after the response, and has been linked to error awareness and response evaluation (20). The N2 is a negative-going, stimulus-locked ERP that occurs 250 to 350 ms after a target, is largest when the stimulus cues multiple conflicting responses, and is widely accepted as reflecting conflict monitoring and detection (19,21). Highlighting their role in higher-order control, these components are frontocentrally distributed and have been source localized to different subregions within the anterior cingulate cortex (20,21), a region known to be involved in cognitive control (22, 23, 24). In accordance with the proposal of inefficient cognitive control in ADHD (14), all 3 components have been found to show reduced amplitude in the condition (16,25), although findings have been far from unanimous and appear most consistent for the Pe (17).

Importantly, these ERP components likely reflect time- and phase-locked aspects of oscillatory activity in the theta range (4–8 Hz) over medial frontal recording sites (known as midfrontal or frontal midline theta [FMΘ]) (7,26, 27, 28, 29). FMΘ activity, in turn, is a robust correlate of cognitive control processes (30, 31, 32). Cross-trial variability in the phase of such activity during cognitive control tasks has been consistently linked to variability in behavioral performance as measured by reaction time variability (RTV) (33, 34, 35, 36, 37). RTV has also consistently been found to be increased in people with ADHD (38, 39, 40, 41). Accordingly, using genetic multivariate model fitting, McLoughlin *et al.* (27) found that theta phase variability was related to both RTV in a conflict task and ADHD status in an adolescent sample, both phenotypically and genetically, suggesting that people with ADHD implement control-related signals in a less temporally coordinated fashion, and this dysregulation is partly genetically determined.

Here, we aimed to extend the findings of McLoughlin *et al.* (27) in a larger sample of young adults using the same Eriksen flanker task, which is known to elicit strong theta activity and consistent theta-related ERPs (8,27,42). In the current study, we measured phase coherence of theta using intertrial phase coherence (ITC), a more widely used measure of phase variability that captures the similarity of phases at a given time point across trials (43). Our first aim for this study was to investigate whether we could replicate the phenotypic and genetic overlap between variability in FMΘ and RTV in this independent sample. Our second aim was to extend the original findings by using longitudinal data to investigate how both childhood and adulthood ADHD-related symptoms were related to electroencephalogram (EEG)-based indices of cognitive control in adulthood, including theta-related ERPs, both phenotypically and genetically. This allowed us to determine the stability of the genetic overlap between FMΘ measures and ADHD across time. The current sample also enabled us to conduct analyses of the genetic and phenotypic overlap between measures of cognitive control and autism spectrum disorder (ASD). ASD shows high rates of co-occurrence with ADHD (44,45), and the results of twin, family, and genetic studies suggest common etiological pathways (46, 47, 48). Here, we examined whether the genetic and phenotypic structure of cognitive control alterations shared common features across the two conditions. Finally, in addition to ITC, we investigated the relationship between ADHD and ASD and the N2, the ERN, and the Pe. As a comparison to theta ITC, we also examined theta power in the same condition and time range to assess whether findings were specific to variability in responding or more general to FMΘ.

## Methods and Materials

This publication is part of the analysis that was preregistered at the start of the project in https://doi.org/10.17605/OSF.IO/AWC5V.

### Sample

The sample consisted of 119 monozygotic (MZ) and 164 dizygotic (DZ) twin pairs (*N* = 566; 271 males). Participants were recruited from the TEDS (Twins Early Development Study) cohort (49), and the sample was enriched for high levels of ADHD and autistic traits based on childhood and adolescent measures. Childhood questionnaire measures were recorded at age 11.09 ± 0.74 years, and the young adulthood diagnostic and cognitive EEG measures were recorded at age 22.44 ± 0.96 years. During young adulthood, 111 participants met criteria for ADHD (based on the Diagnostic Interview for ADHD in Adults 2.0), 47 met criteria for ASD (based on the Autism Diagnostic Observation Schedule, Second Edition), and 16 met criteria for both ADHD and ASD. More detail on the study sample can be found in Aydin *et al.* (48). Ethical approval for the study was received from the King’s College London Psychiatry, Nursing and Midwifery Research Ethics Subcommittee (RESCMR-16/17-2673), and all participants signed informed consent forms prior to participation.

### Adult Diagnostic Interviews

The Diagnostic Interview for ADHD in Adults 2.0 is a semistructured interview that was conducted by a trained investigator to assess ADHD symptoms (50). We used the DSM-5 diagnostic criteria for adult ADHD, which requires 5 or more symptoms of inattention and/or hyperactivity/impulsivity that cause problems in more than one life domain, with recall of childhood onset of symptoms before age 12 (51). The Autism Diagnostic Observation Schedule is a semistructured assessment that allows observations of social and communication behaviors relevant to the diagnosis of autism. We used module 4, which is for adolescents and adults with fluent speech (52). Both the Autism Diagnostic Observation Schedule, Second Edition, and Diagnostic Interview for ADHD in Adults 2.0 were evaluated by trained investigators [see Capp *et al.* (53) for details]. Two questionnaires related to adulthood ADHD and ASD, the Barkley Adult ADHD Rating Scale-IV (BAARS) and the Social Responsiveness Scale, Second Edition (SRS), were also administered to participants. Definitions of BAARS and SRS and related findings are provided in the Supplement.

### Childhood Questionnaires

The Childhood Asperger Syndrome Test (CAST) is a 31-item questionnaire measure of autism-related behaviors and traits (parent reported) (54,55). We used the overall scale (ranging from 0 to 30). The Conners’ Parent Rating Scale, Revised (CPRS) is an 18-item questionnaire measure of ADHD-related traits (parent reported). We used the overall scale, which has a maximum score of 54 (56). The Strengths and Difficulties Questionnaire (SDQ) is a questionnaire measure of emotional and behavioral difficulties (child reported). We used the total behavioral problems score (20 items, maximum score of 40) (57).

### EEG Acquisition and Cognitive Task

**Figure 1:**
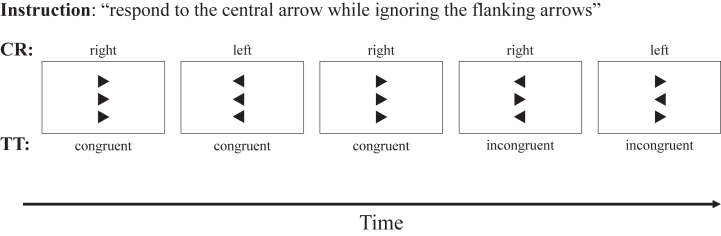
The task is identical to the Eriksen flanker paradigm used by us and colleagues in previous studies (27, 42) and consisted of 10 blocks of 40 trials delivered using PsychoPy software (79). Two flankers (black arrowheads above and below the position of a fixation mark) were presented for 100 ms before the central target black arrowhead appeared for an additional 150 ms. Participants had to press a response button with the index finger of the hand (left or right) corresponding to the direction indicated by the target arrow (left or right). On congruent trials, flanker and target arrowheads pointed in the same direction; on incongruent trials, they pointed in opposite directions. Cognitive performance measures were target reaction time (mean response latency in ms after target onset) and intraindividual variability in reaction time (standard deviation of response latency) in the incongruent correct trials. CR, correct response; TT trial type.

The data were acquired as part of IDEAS (Individual Differences in EEG in young Adults Study), in which EEG data from 4 cognitive tasks and resting state were measured for each participant in one session (53). A 64-channel wireless EEG system with Ag/AgCl electrodes was used for acquisition at 500 Hz sampling rate (Cognionics). The tasks were delivered in counterbalanced order across the whole sample, but the order was kept constant within each twin pair (task order did not have a significant effect on measures). EEG and RT measures from the arrow flanker task were analyzed in this study (see Figure 1 for task description) (27,42).

### EEG Processing and Analysis

EEG preprocessing and analysis were performed using EEGLAB and custom MATLAB scripts (version 2019a; The MathWorks, Inc.) (58). Raw EEG data were filtered 1 to 30 Hz and resampled to 256 Hz. Channels with a correlation of less than 0.4 with their neighbors or above 75 μV (absolute value) more than 15% of the time were marked as bad channels. For more information on data preprocessing and trial epochs, see the Supplement. Trials that exceeded an amplitude threshold (±100 μV) were removed, and participants with at least 20 clean epochs for the condition of interest were used in further analysis (see Table S1 for the number of participants with valid data for each ERP).

Peak amplitudes and latencies were calculated for ERN, Pe, and N2 components, as detailed below. ERN and Pe components were selected from incongruent incorrect trials as the negativity or positivity at the maximal channel (FCz) after the commission of errors (0–150 ms for ERN, 100–350 ms for Pe) (60). N2 components were calculated from congruent and incongruent correct trials as the maximum negativity at the FCz channel 250 to 450 ms after the target stimuli (59). N2 amplitude and latency variables used in the statistical analysis were the difference between congruent and incongruent conditions to reflect the effects of congruency (25,42). Findings related to latencies are provided in the Supplement.

Power and ITC in the theta band were calculated using incongruent correct trials because this condition reflects the greatest conflict and need for cognitive control. The theta frequency used to compute the ITC was calculated as a mean over the individual participant theta frequencies. The individual participant theta frequency was determined as the frequency with maximum ITC within the 5 to 9 Hz band. No significant group difference was found for ITC theta frequencies (*p* = .81); accordingly, the global mean frequency (6.9 Hz) was used to determine individual ITC. A single mean theta was used as opposed to the individual ITC maxima to reduce the number of estimated parameters. For further information on calculation of ITC, see the Supplement. Theta power was calculated using the newtimef function in EEGLAB from the appearance of the target stimulus to 500 ms, with baseline selected as −400 to −100 ms.

### Twin Modeling and Statistical Analysis

Based on the principles that MZ twins share 100% and DZ twins share 50% of their genetic influences while MZ and DZ twins share common environmental factors equally (60), we applied genetic multivariate liability threshold models. These models considered adulthood ADHD and ASD conditions as dichotomous and used the MZ:DZ ratio of the cross-twin within-trait correlations to decompose the variation in the EEG traits into standardized additive genetic variance (a^2^), common environment variance (c^2^), and unique environment variance and measurement error (e^2^). The zero-order correlation between traits (phenotypic correlation [*R*_ph_]) was calculated, and the MZ:DZ ratio of the cross-trait, cross-twin correlations was used to decompose the covariation between traits (e.g., ADHD and theta measure) into genetic (*R*_a_) and common environment (*R*_c_) and unique environment (*R*_e_) correlations. A genetic correlation of 1.0 implies that all additive genetic influences on one trait (e.g., ADHD) also impact the second trait (e.g., theta), whereas a correlation of 0 implies that these influences are independent. Because the sample included a higher ratio of participants who met ADHD or ASD diagnostic criteria than the general population, we performed corrections to the standard twin model [detailed in the Supplement and in a previous study (48)]. Twin modeling was performed using structural equation models in OpenMx (61), and likelihood-based asymmetrical 95% confidence intervals were estimated for all parameters.

Adulthood ADHD or ASD were always included in the model to avoid bias due to sample selection (48). Owing to sample size constraints, our statistical models necessarily had to contain subsets of covariates. Thus, we ran 3 sets of multivariate models. First, we investigated the ADHD/ASD research diagnosis, CAST, CPRS, and SDQ with the EEG/reaction time variables. Second, we ran models with CAST, CPRS, and SDQ to examine the covariance between the research diagnoses and these measures (see Supplement). Finally, we ran models with the SRS and BAARS (see Supplement). All models were fitted on raw data of the whole sample, including typically developed individuals.

## Results

### Within-Trait Genetic Model Fitting

**Figure 2:**
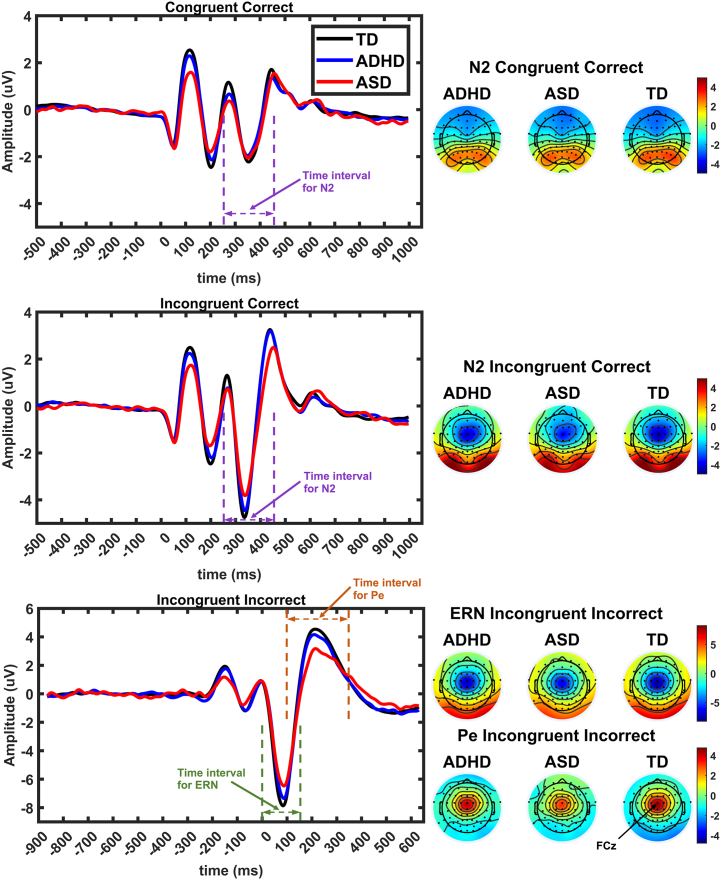
Event-related potentials for typically developing (TD), attention-deficit/hyperactivity disorder (ADHD), and autism spectrum disorder (ASD) groups for the 3 trial types of the arrow flanker task. The topographies for the N2, error-related negativity (ERN), and error positivity (Pe) components are shown in the right panel, with black dots indicating electrode positions. Time intervals used to find the peaks for each component are indicated in waveform panels. The position of the FCz electrode is indicated on the topography map of the Pe.

The grand average ERN and N2 for each group and the corresponding scalp topographic maps are shown in Figure 2. The genetic model fitting indicated moderate to strong significant genetic contributions (a^2^) to the total variance for all questionnaires and EEG and RT measures (Table 1). Common environment contributions (c^2^) were not significant, whereas nonshared environment was significant for all measures. The genetic contribution to the childhood CPRS questionnaire was significantly higher than that for all other measures: 0.90 (95% CI, 0.84–0.92) (Table 1). Childhood CAST had the second-highest point estimate (0.73), but it was not significantly greater than the genetic contributions to ITC and theta power (Table 1). The highest genetic correlation point estimate of the EEG variables was for theta power (0.70) (Table 1) followed by the ERN (0.56) (Table 1). The genetic model fitting results for SRS, BAARS, and ERP latencies are reported in Table S2.

**Table 1:** Standardized Estimates of Genetic (a^2^), Common (c^2^), and Unique (e^2^) Environment Contributions to Variance of Childhood ADHD and Autism Measures and Adulthood EEG and RT Measures

Measures	a^2^ (95% CI)	c^2^ (95% CI)	e^2^ (95% CI)
Childhood CAST	0.73^a^ (0.65 to 0.79)	0.01 (0.00 to 0.07)	0.26^a^ (0.21 to 0.32)
Childhood CPRS	0.90^a^ (0.84 to 0.92)	0.01 (0.00 to 0.07)	0.10^a^ (0.08 to 0.12)
Childhood SDQ	0.51^a^ (0.27 to 0.68)	0.09 (0.00 to 0.28)	0.40^a^ (0.32 to 0.49)
ITC	0.33^a^ (0.08 to 0.66)	0.30 (0.00 to 0.50)	0.37^a^ (0.28 to 0.50)
Amplitude of ERN	0.56^a^ (0.46 to 0.64)	0.00 (0.00 to 0.06)	0.44^a^ (0.36 to 0.54)
Amplitude of Pe	0.19^a^ (0.04 to 0.47)	0.19 (0.00 to 0.33)	0.62^a^ (0.51 to 0.73)
Amplitude of N2	0.41^a^ (0.16 to 0.51)	0.00 (0.00 to 0.18)	0.59^a^ (0.49 to 0.71)
Theta Power	0.70^a^ (0.57 to 0.77)	0.00 (0.00 to 0.12)	0.30^a^ (0.23 to 0.36)
RTV	0.32^a^ (0.18 to 0.45)	0.00 (0.00 to 0.10)	0.68^a^ (0.55 to 0.82)
RTM	0.55^a^ (0.42 to 0.62)	0.00 (0.00 to 0.14)	0.45^a^ (0.38 to 0.58)

ADHD, attention-deficit/hyperactivity disorder; CAST, Childhood Asperger Syndrome Test; CPRS, Conners' Parent Rating Scale; EEG, electroencephalogram; ERN, error-related negativity; ITC, intertrial coherence; Pe, error positivity; RTM, reaction time mean; RTV, reaction time variability; SDQ, Strengths and Difficulties Questionnaire.

a Significant estimates.

### Cross-Trait Genetic Model Fitting

#### ITC and RT Measures

We found that theta ITC had a large phenotypic and genetic overlap with RTV (Table 2). Notably, our results indicated that more than two-thirds of the genetic influences on RTV were shared with theta ITC. The shared genetic influences between RTV and ITC were significantly different from the (nonsignificant) shared genetic influences between RT mean (RTM) and ITC, indicating a specific genetic association between ITC and RTV. There was also a significant phenotypic association between ITC and RTM, and RTV and RTM shared substantial phenotypic and genetic associations with each other (Table 2).

**Table 2:** Phenotypic (*R*_ph_) and Genetic (*R*_a_) Correlations Between RTV, RTM, and ITC

Measures	*R*_ph_ (95% CI)	*R*_a_ (95% CI)
RTV-ITC	−0.39^a^ (−0.46 to −0.31)	−0.68^a^ (−1.00 to −0.21)
RTM-ITC	−0.26^a^ (−0.34 to −0.17)	−0.34 (−0.84 to 0.20)
RTV-RTM	0.58^a^ (0.52 to 0.64)	0.65^a^ (0.19 to 0.87)

Common and unique environment correlations are reported in Table S3.

ITC, intertrial coherence; RTM, reaction time mean; RTV, reaction time variability.

a Significant estimates.

RTV showed significant phenotypic and genetic associations with both childhood measures and adult diagnoses of ADHD and ASD, as well as a significant phenotypic correlation with the SDQ. In the case of adult ASD, the genetic overlap with RTV was substantial and more than twice the point estimate for the genetic correlation with ADHD (although the confidence intervals overlapped, indicating that there was no significant difference). Relationships between RTM and clinical measures, although significant, were more moderate in size (Table 3).

**Table 3:** Phenotypic (*R*_ph_) and Genetic (*R***_a_**) Correlations Between ITC, Theta Power, and RT Variables and Childhood Symptom Questionnaires (CAST, CPRS, and SDQ) and Adulthood ADHD and ASD

Measures	Childhood CAST		Childhood CPRS		Childhood SDQ		Adulthood ADHD		Adulthood ASD	
	*R*_ph_ (95% CI)	*R*_a_ (95% CI)	*R*_ph_ (95% CI)	*R*_a_ (95% CI)	*R*_ph_ (95% CI)	*R*_a_ (95% CI)	*R*_ph_ (95% CI)	*R*_a_ (95% CI)	*R*_ph_ (95% CI)	*R*_a_ (95% CI)
ITC	−0.07 (−0.17 to 0.04)	−0.08 (−0.41 to 0.30)	−0.23^a^ (−0.32 to −0.12)	−0.44^a^(−0.92 to −0.28)	−0.13^a^ (−0.23 to −0.02)	−0.20 (−0.80 to 0.39)	−0.19^a^ (−0.30 to −0.05)	−0.33^a^ (−0.77 to −0.03)	−0.18^a^(−0.33 to −0.01)	−0.31 (−0.79 to 0.10)
Theta Power	−0.04 (−0.12 to 0.03)	−0.06 (−0.17 to 0.07)	−0.11^a^ (−0.18 to −0.03)	−0.13^a^(−0.23 to −0.03)	−0.02 (−0.09 to 0.05)	−0.06^a^ (−0.26 to −0.04)	−0.05 (−0.14 to 0.06)	0.03 (−0.13 to 0.18)	−0.07 (−0.24 to 0.11)	−0.03 (−0.32 to 0.25)
RTV	0.13^a^ (0.05 to 0.21)	0.34^a^ (0.12 to 0.57)	0.20^a^ (0.11 to 0.28)	0.34^a^(0.15 to 0.51)	0.13^a^ (0.05 to 0.22)	0.13 (−0.41 to 0.57)	0.17^a^ (0.07 to 0.27)	0.27^a^ (0.05 to 0.56)	0.32^a^ (0.16 to 0.45)	0.61^a^ (0.17 to 0.98)
RTM	0.04 (−0.04 to 0.12)	0.05 (−0.09 to 0.23)	0.08 (0.00 to 0.16)	0.13^a^(0.01 to 0.25)	0.07 (−0.02 to 0.17)	0.07 (−0.39 to 0.47)	0.05^a^ (0.05 to 0.15)	0.14 (−0.07 to 0.33)	0.18^a^ (0.02 to 0.32)	−0.01 (−0.32 to 0.32)

Common and unique environment correlations are reported in Table S4.

ADHD, attention-deficit/hyperactivity disorder; ASD, autism spectrum disorder; CAST, Childhood Asperger Syndrome Test; Corr, correlation; CPRS, Conners’ Parent Rating Scale; ITC, intertrial coherence; RT, reaction time; RTM, RT mean; RTV, RT variability; SDQ, Strengths and Difficulties Questionnaire.

a Significant estimates.

Similarly strong relationships were found for neural measures of variability. Lower theta ITC was significantly phenotypically and genetically correlated with both childhood measures and adult diagnosis of ADHD, with additional phenotypic—but not genetic—correlations with the SDQ and adult ASD (Table 3). The genetic relationship between theta ITC and childhood ADHD symptoms was independent of theta power.

#### ERP Variables

The amplitudes of the ERN and N2 were significantly correlated with childhood ADHD symptoms, but not with adult diagnoses, at both the phenotypic and genetic levels. Phenotypically, there was a significant association between ERN amplitude and childhood ASD symptoms, but there was no genetic overlap. Pe amplitude was significantly phenotypically associated with all diagnostic and questionnaire measures (Table 4). Moreover, the Pe exhibited significant genetic correlations with childhood ADHD and ASD and with adult diagnoses. In the case of CAST scores and adult ASD diagnosis, this was substantial, showing around two-thirds shared genetic variance. The negative genetic correlations indicate that the genes that affect ADHD and ASD symptoms in both childhood and adulthood also affect lower Pe amplitude measured in adulthood.

**Table 4:** Phenotypic (*R*_ph_) and Genetic (*R*_a_) Correlations Between Event-Related Potential Variables and Childhood Symptom Questionnaires (CAST, CPRS, and SDQ) and Adulthood ADHD and ASD

Measures	Childhood CAST		Childhood CPRS		Childhood SDQ		Adulthood ADHD		Adulthood ASD	
	*R*_ph_ (95% CI)	*R*_a_ (95% CI)	*R*_ph_ (95% CI)	*R*_a_ (95% CI	*R*_ph_ (95% CI)	*R*_a_ (95% CI)	*R*_ph_ (95% CI)	*R*_a_ (95% CI)	*R*_ph_ (95% CI)	*R*_a_ (95% CI)
Amplitude of ERN	−0.11^a^ (−0.18 to −0.04)	−0.13 (−0.26 to 0.00)	−0.14^a^ (−0.21 to −0.07)	−0.17^a^ (−0.28 to −0.06)	−0.05 (−0.14 to 0.02)	0.06 (−0.19 to 0.37)	−0.06 (−0.15 to 0.03)	−0.05 (−0.22 to 0.11)	−0.14 (−0.29 to 0.03)	−0.24 (−0.55 to 0.09)
Amplitude of Pe	−0.19^a^ (−0.26 to −0.12)	−0.68^a^ (−1.00 to −0.28)	−0.18^a^ (−0.25 to −0.10)	−0.44^a^ (−0.97 to −0.14)	−0.10^a^ (−0.17 to −0.02)	−0.12 (−1.00 to 0.55)	−0.09^a^ (−0.19 to −0.01)	−0.36^a^ (−0.82 to −0.04)	−0.28^a^ (−0.43 to −0.11)	−0.65^a^ (−1.00 to −0.11)
Amplitude of N2	−0.04 (−0.11 to 0.04)	−0.05 (−0.26 to 0.12)	−0.10^a^ (−0.17 to −0.02)	−0.16^a^ (−0.37 to −0.01)	−0.03 (−0.10 to 0.03)	−0.07 (−0.42 to 0.20)	−0.08 (−0.17 to 0.01)	0.03 (−0.17 to 0.24)	−0.08 (−0.24 to 0.08)	−0.15 (−0.66 to 0.23)

Common and unique environmental correlation are reported in Table S4. *R*_ph_ and *R*_a_ for latencies are reported in Table S5.

ADHD, attention-deficit/hyperactivity disorder; ASD, autism spectrum disorder; CAST, Childhood Asperger Syndrome Test; Corr, correlation; CPRS, Conners' Parent Rating Scale; ERN, error-related negativity; Pe, error positivity; SDQ, Strengths and Difficulties Questionnaire.

a Significant estimates.

#### Questionnaires and Diagnosis

Cross-trait genetic model findings between childhood (CAST, CPRS, SDQ) and adulthood questionnaires (BAARS, SRS) and adulthood conditions (ADHD, ASD) are reported in Tables S6–S8.

## Discussion

Results from these multivariate twin analyses indicate that specific FMΘ-related measures recorded in young adulthood (22 years of age) share genetic overlap with concurrent ADHD and ASD diagnosis and with ADHD and ASD symptoms previously measured in the same individuals during middle childhood (11 years of age). ADHD measures from both time points were most strongly related to the Pe and to FMΘ ITC (phase variability). The latter result extends our previous finding showing a phenotypic and genetic overlap between ADHD measured at age 14 and a concurrent measure of FMΘ phase variability across trials (27).

In a further extension of our previous findings, we found that instability in behavioral responses, indexed by RTV, shares genetic and phenotypic overlap with ADHD at both time points. Our almost precisely identical replication of the phenotypic and genetic overlap between variability in FMΘ (indexed here by ITC) and RTV is critical to the interpretation of these findings. Our previous analysis of FMΘ phase variability and RTV in adolescents indicated a genetic overlap of 0.66 (27), and in the current investigation, we found a genetic overlap of 0.68 in young adulthood. These findings provide strong additional evidence that dysregulation of theta signaling in ADHD may be a mechanism for failure to implement and optimize task-relevant responding in the disorder (7).

The negative phenotypic and genetic overlap between the Pe and childhood symptoms of ADHD and the diagnosis in adulthood is consistent with previous findings indicating that the Pe is typically reduced in ADHD (17). While the phenotypic overlap with ADHD diagnosis in adulthood was small yet significant (−0.09), the phenotypic overlap with ASD diagnosis was substantially stronger (−0.28). This contrasts with the findings in relation to childhood symptoms, where there was similar phenotypic association between the Pe and ADHD (−0.19) and ASD (−0.18) symptoms. A novel finding here was the moderate to high genetic correlations between the Pe and both childhood ASD symptoms and adult ASD diagnosis. This is consistent with some previous findings indicating the relevance of the Pe to ASD (62,63); however, not all studies agree, with the Pe also having been found to be unaffected in ASD (64).

A possible reason for the discrepancy between the strong findings in the current study and the less consistent previous findings in the ASD literature is the task-dependent nature of the Pe (65). Indeed, in the current study, we found that the Pe was more frontal and earlier than has been found in relation to other paradigms. This is consistent with studies that have shown an early frontocentral component that diverges from a later centroparietal deflection that emerges around 300 to 500 ms after error onset (20,66,67). The later Pe is well established as related to error awareness because it is only observed for consciously detected errors (68) and has been proposed to represent a P3-like facilitation of information processing modulated by subcortical arousal systems (20). The functional relevance of the early frontocentral Pe has not been not well established, but the occurrence of the Pe directly after the theta oscillation in our data suggests a possible relationship to FMΘ or to the same process that underlies the theta oscillation. Future research could aim to decompose the source of these signals and further investigate the impact of task design parameters to enable more accurate interpretation of Pe-related findings in these disorders.

For the other FMΘ-related ERPs, the ERN and N2, the findings indicate phenotypic and genetic overlap with childhood ADHD symptoms and a small phenotypic overlap with childhood ASD symptoms but no overlap with either adult diagnosis. These results are in line with inconsistent findings in relation to the N2 and ERN and ADHD and ASD (15,17,69,70). Similarly, FMΘ power showed limited overlap with ADHD or ASD across the measures, with no significant relationships evident with either adult diagnosis. A small genetic overlap between theta power and childhood ADHD symptoms was significantly lower than the genetic overlap between theta ITC and childhood symptoms. Overall, the findings indicate that variability in FMΘ phase (rather than power) is relevant to ADHD.

A novel aspect of our study is the ability to take a longitudinal perspective on the relationship between contemporary measurements of childhood ADHD and ASD symptoms and theta-related EEG measures on the same individuals measured 11 years later during young adulthood. Our findings indicate strong and significant shared phenotypic and genetic variation between several measures and childhood symptoms. Specifically, in the case of the CPRS (childhood ADHD symptoms), significant phenotypic and genetic overlap emerged for all theta-related measures and RTV in adulthood. The genetic overlap was moderate for RTV, ITC, and the Pe. This indicates that, in the case of the Pe and ITC in particular, almost one-half of the genetic variance driving childhood ADHD symptoms is driving differences in FMΘ indices of cognitive control in young adulthood. The broad relationship between the CPRS and differences in cognitive control during young adulthood may highlight the importance of childhood symptoms in predicting later functional impacts on cognition, possibly even more strongly than a later diagnosis of the condition. This is particularly important in the context of suggestions that the prevalence of ADHD in adulthood is an underestimate: 2.5% versus 5% in childhood (71). Clinical observations indicate that the symptoms manifest differently with development into adulthood. For example, the hyperactivity of childhood ADHD manifests more as a sense of internal restlessness in adults (72). Furthermore, some adults with ADHD may not present with a typical pattern of functional difficulties in their daily life so that, similar to ASD (73), behavioral characteristics associated with the condition can be masked by adaptive or compensatory skills (74). It could be that these compensatory skills cannot mask underlying fundamental alterations in cognitive control, which may thus be indicative of the ongoing impact of the condition on those with childhood symptoms.

While the ASD measures in childhood (CAST) did not share the same broad overlap with the EEG measures, a compelling finding was the large shared genetic variance (more than two-thirds) between these symptoms in childhood and the Pe in young adulthood (matched almost precisely to the shared genetic variance between the Pe and ASD diagnosis in young adulthood). These findings indicate that the etiological factors that drive childhood symptoms affect specific functional alterations in cognitive control in adulthood and add convincing evidence to the neurobiological validity of these measures in the context of these disorders. Both the CAST and the CPRS were based on parent reports, whereas the SDQ was based on self-report, which could explain the relatively lower heritability of the SDQ and its limited overlap with EEG measures (75,76). The lack of evidence for significant common (shared) environment contributions is consistent with many other twin studies. Multiple interpretations have been suggested, such as that most environments may be nonshared because environments are perceived and experienced differently by twins who grow up in the same family (77,78).

While our sample is large for a twin study with EEG measures, it is possible that a larger study would provide greater clarity on the relative strengths of the relationships. In most cases, the confidence intervals for the estimates overlapped, and thus it was not possible to definitively identify which of the relationships were stronger. In addition, the necessary use of a biometric genetic model that had to include either ADHD or ASD precluded the estimate of shared variance between the two disorders. However, the models did allow us to estimate which FMΘ measures were specific and shared between the two disorders, with strong evidence that ITC was specific to ADHD whereas the Pe and RTV are shared alterations across the two disorders in relation to childhood symptoms and adult diagnoses. In addition, our models did not allow us to test causality. It could be true that the shared genetic variance between, for example, RTV and FMΘ ITC is due to pleiotropic effects or other factors that influence dysregulation in both brain signaling and behavior.

In summary, in a large, rigorously collected twin sample with measures obtained in young adulthood, in addition to previously obtained measurements of childhood ADHD and ASD, a multivariate twin analysis indicated the importance of FMΘ-related and RT measures in the neurobiology of the conditions. We extended our previous finding of the overlap between ADHD and variability in theta signaling in the frontal midline cortex and further precisely replicated our findings of shared genetic variance between this theta variability and variability in responding as indexed by RTV (27). While we had previously found that sources identified by independent component analysis showed the strongest relationships to the disorder and response behavior, the current data indicate that the more easily obtained measure of ITC in sensor space is also effective for detecting these alterations in theta, which could have more impact on the development of clinically relevant biomarkers.

Adequate cognitive control is critical for optimal daily life functioning, and our finding that childhood symptoms predicted suboptimal cognitive control in young adulthood highlights the need for broader investigations of functional impairment in individuals who had symptoms or diagnosis of these conditions in childhood and of whether or not such individuals retain the diagnosis in adulthood. Future research should investigate how these measures impact quality of life and broader measures of functional impairment, including measures of life skills, work and school performance, and social interactions (48).

## Supplementary Material

Supplementary Material

Key Resources Table
