# Supplementary material for: Genetic Overlap Between Midfrontal Theta Signals and Attention-Deficit/Hyperactivity Disorder and Autism Spectrum Disorder in a Longitudinal Twin Cohort
Source: Biol Psychiatry. 2023 Nov 15;94(10):823–32. doi: 10.1016/j.biopsych.2023.05.006 (PMC10769884; doi:10.1016/j.biopsych.2023.05.006)
Supplement: Supplementary Material [file mmc1.pdf]

# SUPPLEMENTARY INFORMATION

# Genetic Overlap Between Midfrontal Theta Signals and Attention-Deficit/Hyperactivity Disorder and Autism Spectrum Disorder in a Longitudinal Twin Cohort

Aydin *et al.*

## EEG processing and analysis

Following average referencing, Adaptive Mixture ICA (AMICA) was used to calculate ICA components (1) using the  nsgportal plug-in on the high performance computing resources available on Neuroscience Gateway (NSG, nsgportal.org) (2). The ICLabel algorithm was used for automatic detection and removal of ICs representing ocular artefacts (3). Continuous data was divided into epochs. Incongruent-incorrect trials were response-locked to incorrect responses with epochs -900 to 600 ms based on the time of the button press. Congruent-correct and incongruent-correct trials were stimulus-locked to correctly answered stimuli with epochs from -500 ms to 1000 ms relative to the onset of the target stimulus. All trials were baseline corrected using baseline  -900 ms to -600 ms for response-locked and -400 ms to -100 ms for stimulus-locked trials.

## Calculation of ITC

ITC was calculated over a window of 3 cycles at the mean frequency of 6.9 Hz (435 ms) starting 100 ms after the flanker stimulus, simultaneous with the onset of the central target stimulus, and ending before the response on average. The measure used for ITC is equivalent to the Phase Locking Value (PLV) defined in Lachaux et al (4), where the phase locking value is calculated between the trial central midline waveform and the target stimulus impulse signal, in the 3 cycle window starting 100 ms after the flanker stimulus to account for perceptual delay.

The formula for computing the complex normalized phase associated with an individual trial $j$ is,

$$P_j = \sum_{k=1}^{T} s(k_{100} + k)e^{i2\pi fk/T}$$

Where $s_{FCz,j}(k)$ is $j$th trial epoch of the EEG FCz channel time series, $k_{100}$ is the sample corresponding to the central target onset (100 ms after the flanker onset), $e^{i2\pi fk/T}$ is the complex exponential, $f$ is the mean of the participant maximal ITC theta frequencies, $k$ is the sample index, and $T$ is the number of samples in 3 cycles of the mean max ITC theta frequency $f$. Then the ITC for a group of $N$ trials is calculated length (absolute value) of the mean of the normalized complex phasors,

$$ITC = \left| \frac{1}{N} \sum_{j=1}^{N} |P_j| \right|$$

**Questionnaire measures**

In addition to the DIVA and ADOS described in the main manuscript two other questionnaires were included in the analyses:

- *Barkley Adult ADHD Rating Scale-IV (BAARS):* is an empirically developed self-rating scale, based on DSM diagnostic criteria. It is suggested to evaluate the most reliable underlying dimensions of the symptom list for adults (5). Here we used the overall scale ranging from 18 to 72.

- *Social Responsiveness Scale 2nd edition (SRS):* is a self-reported scale measuring deficit in social behaviour associated with autism and their severity (6). Here we used the raw sum scores ranging from 0 to 195.

**Twin Modelling**

Since the sample was selected on affection status of either ADHD or autism, some corrections are needed within the standard twin model (7). First, one of the selection variables always needs to be included so that bivariate models are the smallest models to be fitted. Second, fixing the thresholds to the population prevalence of 4% for ADHD (8) and 1% for autism (9). The third adjustment is fixing twin correlations and heritability parameters for ADHD and autism to population estimates: $a^2 = 0.76$, $c^2 = 0$, $e^2 = 0.24$, rMZ = 0.76, rDZ = 0.38, using the same estimates for ADHD and autism (9,10). It was necessary to include ADHD and autism variables in each model to avoid bias due to the enriched study sample. Throughout this study we will only refer to the $a^2$, $c^2$ and $e^2$ estimates in the model selected on ADHD because the estimates did not vary whether they were examined in the model with ADHD or autism. Due to the inclusion of the two dichotomous variables (ADHD and ASD), the genetic models were calculated once with each. However, the estimates did not vary; thus we only showed the estimates obtained with ADHD unless Rph, Ra, Rc and Re with ASD are being reported (11).

**Table S1:** The number of participants that have valid diagnostic and questionnaire, EEG and reaction time measures after artefact rejection prior to outlier detection as well as the number of outliers detected for each measure are outlined in the table below. Prior to twin analysis, outliers in EEG and reaction time measures were excluded based on the 2*IQR (interquartile range) criterion.

| Psychological and EEG measures | Number of participants with valid data prior to outlier detection | Number of outliers detected |
|---|---|---|
| **DIVA** | 556 | **-**[1] |
| **ADOS** | 547 | **-**[1] |
| **SRS** | 483 | **-**[1] |
| **BAARS** | 490 | **-**[1] |
| **Childhood CAST** | 508 | **-**[1] |
| **Childhood CPRS** | 510 | **-**[1] |
| **Childhood SDQ** | 508 | **-**[1] |
| **ITC** | 516 | 2 |
| **Amplitude of ERN** | 509 | 2 |
| **Amplitude of Pe** | 509 | 3 |
| **Amplitude of N2** | 516 | 4 |
| **Theta power** | 516 | 6 |
| **RTV** | 516 | 25 |
| **RTM** | 516 | 8 |
| **Latency of ERN** | 509 | 4 |
| **Latency of Pe** | 509 | 0 |
| **Latency of N2** | 516 | 51 |

[1] Outlier detection was only performed for EEG variables and reaction time measures.

**Table S2:** The variances for adulthood questionnaires and latency measures estimated by twin analysis.

| Measures | $a^2$ | $c^2$ | $e^2$ |
|---|---|---|---|
| **Adulthood BAARS** | **0.19**<br>0.09,0.40 | 0.07<br>0.00,0.16 | **0.74**<br>0.60,0.84 |
| **Adulthood SRS** | **0.49**<br>0.35,0.61 | 0.09<br>0.00,0.19 | **0.43**<br>0.35,0.51 |
| **Latency of ERN** | 0.06<br>0.00,0.47 | 0.27<br>0.00,0.41 | **0.67**<br>0.51,0.80 |
| **Latency of Pe** | **0.26**<br>0.14,0.38 | 0.00<br>0.00,0.19 | **0.74**<br>0.62,0.86 |
| **Latency of N2** | **0.24**<br>0.01,0.38 | 0.00<br>0.00,0.20 | **0.75**<br>0.62,0.89 |

[1] 95% confidence intervals (CIs) are included under each estimate and significant estimates are written in bold.

**Table S3:** Rc and Re between RTV, RTM, and ITC.

| | Rc | Re |
|---|---|---|
| **RTV - ITC** | -1.00<br>-1.00,1.00 | -0.14<br>-0.31,0.04 |
| **RTM - ITC** | -1.00<br>-1.00,1.00 | -0.03<br>-0.20,0.14 |
| **RTV - RTM** | 1.00<br>-1.00,1.00 | **0.53**<br>0.39,0.64 |

[1] 95% confidence intervals (CIs) are included under each estimate and significant estimates are written in bold.

**Table S4:** Rc and Re between EEG and behavioural measures and childhood questionnaires (CAST, CPRS, SDQ) and adulthood conditions (ADHD, ASD).

| Measures | Childhood CAST | | Childhood CPRS | | Childhood SDQ | | Adulthood ADHD | | Adulthood ASD | |
|---|---|---|---|---|---|---|---|---|---|---|
| | Rc | Re | Rc | Re | Rc | Re | Rc | Re | Rc | Re |
| **ITC** | 0.89<br>-1.00,1.00 | -0.17<br>-0.35,0.02 | 0.78<br>-1.00,1.00 | -0.02<br>-0.20,0.18 | -0.02<br>-1.00,1.00 | -0.08<br>-0.26,0.12 | -[2] | -0.08<br>-0.39,0.21 | -[2] | -0.04<br>-0.55,0.48 |
| **Amplitude of ERN** | -1.00<br>-1.00,1.00 | -0.08<br>-0.23,0.05 | 0.99<br>-1.00,1.00 | -0.12<br>-0.26,0.03 | -0.99<br>-1.00,1.00 | **-0.18**<br>-0.32,-0.02 | -[2] | -0.07<br>-0.28,0.15 | -[2] | 0.06<br>-0.47,0.59 |
| **Amplitude of Pe** | 1.00<br>-1.00,1.00 | 0.03<br>-0.10,0.16 | 1.00<br>-1.00,1.00 | -0.10<br>-0.23,0.03 | 0.59<br>-1.00,1.00 | **-0.17**<br>-0.30,-0.03 | -[2] | 0.09<br>-0.13,0.30 | -[2] | -0.04<br>-0.52,0.43 |
| **Amplitude of N2** | 0.99<br>-1.00,1.00 | -0.03<br>-0.15,0.09 | 0.92<br>-1.00,1.00 | -0.03<br>-0.17,0.09 | 1.00<br>-1.00,1.00 | -0.08<br>-0.20,0.04 | -[2] | **-0.24**<br>-0.44,-0.05 | -[2] | 0.01<br>-0.46,0.51 |
| **Theta power** | 0.90<br>-0.33,1.00 | -0.01<br>-0.17,0.11 | -0.64<br>-1.00,1.00 | 0.00<br>-0.14,0.15 | 1.00<br>0.99,1.00 | 0.00<br>-0.15,0.12 | -[2] | -0.22<br>-0.45,0.01 | -[2] | -0.16<br>-0.64,0.43 |
| **RTV** | 0.63<br>-1.00,1.00 | -0.07<br>-0.23,0.08 | 0.60<br>-1.00,1.00 | 0.08<br>-0.06,0.25 | 1.00<br>-1.00,1.00 | 0.10<br>-0.06,0.24 | -[2] | 0.09<br>-0.17,0.31 | -[2] | 0.07<br>-0.35,0.48 |
| **RTM** | -0.41<br>-1.00,1.00 | 0.02<br>-0.15,0.17 | 0.06<br>-1.00,1.00 | -0.05<br>-0.20,0.11 | 1.00<br>-1.00,1.00 | 0.07<br>-0.11,0.24 | -[2] | -0.10<br>-0.34,0.17 | -[2] | **0.55**<br>0.01,0.91 |
| **Latency of Ne** | -0.88<br>-1.00,1.00 | 0.17<br>-0.03,0.34 | -1.00<br>-1.00,1.00 | 0.18<br>-0.01,0.35 | 1.00<br>-1.00,1.00 | -0.04<br>-0.21,0.13 | -[2] | 0.21<br>-0.08,0.48 | -[2] | 0.22<br>-0.28,0.70 |
| **Latency of Pe** | 0.86<br>-1.00,1.00 | -0.04<br>-0.18,0.09 | -0.55<br>-1.00,1.00 | -0.10<br>-0.24,0.04 | -1.00<br>-1.00,1.00 | -0.06<br>-0.19,0.08 | -[2] | 0.02<br>-0.19,0.23 | -[2] | 0.35<br>-0.06,0.72 |
| **Latency of N2** | 0.97<br>-0.32,1.00 | 0.10<br>-0.04,0.24 | 0.97<br>-1.00,1.00 | **0.19**<br>0.05,0.34 | 1.00<br>-1.00,1.00 | -0.02<br>-0.16,0.11 | -[2] | -0.02<br>-0.28,0.20 | -[2] | 0.00<br>-0.57,0.53 |

[1] 95% confidence intervals (CIs) are included under each estimate and significant estimates are written in bold.
[2] There is no Rc estimate with adulthood conditions since the $c^2$ values for adulthood conditions were fixed to 0. Please see twin analysis section for details.

**Table S5:** Phenotypic and genetic correlations between ERP latencies and childhood questionnaires (CAST, Conners, SDQ) and adulthood conditions (ADHD, ASD).

| Measures | Childhood CAST | | Childhood CPRS | | Childhood SDQ | | Adulthood ADHD | | Adulthood ASD | |
|---|---|---|---|---|---|---|---|---|---|---|
| | Rph | Ra | Rph | Ra | Rph | Ra | Rph | Ra | Rph | Ra |
| Latency of Ne | 0.03<br>-0.07,0.13 | -0.19<br>-1.00,1.00 | 0.08<br>-0.02,0.19 | 0.27<br>-1.00,1.00 | 0.09<br>-0.01,0.18 | -0.32<br>-1.00,1.00 | **0.19**<br>0.06,0.31 | 0.49<br>-1.00,1.00 | 0.08<br>-0.08,0.24 | -0.03<br>-1.00,1.00 |
| Latency of Pe | 0.07<br>-0.01,0.14 | 0.20<br>-0.01,0.41 | 0.04<br>-0.04,0.11 | 0.13<br>-0.03,0.32 | 0.04<br>-0.04,0.12 | 0.40<br>-0.14,0.99 | 0.03<br>-0.06,0.13 | 0.06<br>-0.19,0.33 | **0.24**<br>0.09,0.39 | 0.20<br>-0.26,0.61 |
| Latency of N2 | **0.13**<br>0.05,0.20 | 0.20<br>-0.22,0.66 | 0.03<br>-0.05,0.10 | -0.06<br>-0.60,0.11 | -0.03<br>-0.11,0.04 | -0.22<br>-0.73,0.97 | 0.09<br>-0.01,0.18 | 0.23<br>-0.04,0.83 | 0.15<br>-0.02,0.32 | 0.36<br>-0.21,1.00 |

[1] 95% confidence intervals (CIs) are included under each estimate and significant estimates are written in bold.

**Table S6:** Phenotypic (Rph) and genetic (Ra) correlations between adulthood questionnaires (BAARS, SRS) and childhood measures (CAST, CPRS, SDQ), and adulthood conditions (ADHD (from DIVA), ASD (from ADOS)).

| Measures | | Childhood CPRS | Childhood CAST | Childhood SDQ | Adulthood ADHD | Adulthood ASD | Adulthood BAARS |
|---|---|---|---|---|---|---|---|
| Adulthood BAARS | Rph | **0.19**<br>0.12,0.25 | **0.11**<br>0.04,0.18 | **0.17**<br>0.11,0.25 | **0.51**<br>0.44,0.58 | 0.08<br>-0.10,0.25 | - |
| | Ra | **0.30**<br>0.05,0.56 | 0.21<br>-0.11,0.51 | **0.64**<br>0.14,1.00 | **0.90**<br>0.55,1.00 | **0.71**<br>0.09,1.00 | - |
| Adulthood SRS | Rph | **0.35**<br>0.29,0.42 | **0.33**<br>0.27,0.40 | **0.38**<br>0.32.0.45 | **0.47**<br>0.39,0.54 | **0.54**<br>0.40,0.65 | **0.33**<br>0.27,0.40 |
| | Ra | **0.48**<br>0.38,0.62 | **0.51**<br>0.36,0.67 | **0.61**<br>0.38,0.84 | **0.65**<br>0.49,0.81 | **0.62**<br>0.36,0.90 | **0.85**<br>0.31,1.00 |

[1] 95% confidence intervals (CIs) are included under each estimate and significant estimates are written in bold.

**Table S7:** Phenotypic (Rph) and genetic (Ra) correlations between childhood symptom questionnaires (CAST, CPRS and SDQ) and adulthood ADHD and ASD.

| Measures | | Childhood CPRS | Childhood CAST | Childhood SDQ |
|---|---|---|---|---|
| Adulthood ADHD | Rph | **0.44**<br>0.35,0.52 | **0.21**<br>0.12,0.30 | **0.35**<br>0.22,0.43 |
| | Ra | **0.54**<br>0.41,0.63 | **0.30**<br>0.16,0.43 | **0.74**<br>0.50,1.00 |
| Adulthood ASD | Rph | **0.54**<br>0.38,0.66 | **0.62**<br>0.51,0.72 | **0.43**<br>0.28,0.57 |
| | Ra | **0.62**<br>0.42,0.78 | **0.80**<br>0.61,0.95 | **0.65**<br>0.31,1.00 |
| Childhood SDQ | Rph | **0.53**<br>0.48,0.57 | **0.39**<br>0.34,0.44 | - |
| | Ra | **0.73**<br>0.62,0.90 | **0.62**<br>0.47,0.81 | - |
| Childhood CAST | Rph | **0.60**<br>0.55,0.64 | - | - |
| | Ra | **0.70**<br>0.64,0.76 | - | - |

[1] 95% confidence intervals (CIs) are included under each estimate and significant estimates are written in bold.

**Table S8:** Rc and Re correlations between childhood measures (CAST, CPRS, SDQ), adulthood questionnaires (BAARS, SRS) and adulthood conditions (ADHD, ASD).

| Measures | | Childhood CPRS | Childhood CAST | Childhood SDQ | Adulthood BAARS | Adulthood SRS |
|---|---|---|---|---|---|---|
| **Adulthood ADHD** | **Rc** | -[2] | -[2] | -[2] | -[2] | -[2] |
| | **Re** | 0.02<br>-0.18,0.24 | -0.03<br>-0.21,0.15 | **-0.33**<br>-0.52,-0.12 | **0.39**<br>0.21,0.55 | **0.27**<br>0.09,0.48 |
| **Adulthood ASD** | **Rc** | -[2] | -[2] | -[2] | -[2] | -[2] |
| | **Re** | 0.17<br>-0.33,0.61 | 0.13<br>-0.25,0.49 | 0.12<br>-0.31,0.55 | -0.44<br>-0.83,0.05 | **0.49**<br>0.41,0.57 |
| **Adulthood SRS** | **Rc** | -1.00<br>-1.00,1.00 | -0.95<br>-1.00,1.00 | 0.59<br>-1.00,1.00 | -0.83<br>-1.00,1.00 | - |
| | **Re** | 0.08<br>-0.07,0.20 | -1.00<br>-1.00,1.00 | 0.07<br>-0.06,0.22 | **0.19**<br>0.06,0.31 | - |
| **Adulthood BAARS** | **Rc** | 1.00<br>-1.00,1.00 | 0.97<br>-1.00,1.00 | -0.04<br>-1.00,1.00 | - | - |
| | **Re** | **0.17**<br>0.03,0.30 | 0.04<br>-0.10,0.20 | 0.10<br>-1.00,1.00 | - | - |
| **Childhood SDQ** | **Rc** | 1.00<br>-1.00,1.00 | -1.00<br>-1.00,1.00 | - | - | - |
| | **Re** | 0.10<br>0.00,0.23 | 0.11<br>-0.01,0.23 | - | - | - |
| **Childhood CAST** | **Rc** | 1.00<br>-1.00,1.00 | - | - | - | - |
| | **Re** | 0.13<br>-0.01,0.26 | - | - | - | - |

[1] 95% confidence intervals (CIs) are included under each estimate and significant estimates are written in bold.

[2] There is no Rc estimate with adulthood conditions since the $c^2$ values for adulthood conditions were fixed to 0. Please see twin analysis section for details.

**Supplementary References**

1. Palmer JA, Kreutz-Delgado K, Makeig S (2011): AMICA: An Adaptive Mixture of Independent Component Analyzers with Shared Components. 15.

2. Martínez-Cancino R, Delorme A, Truong D, Artoni F, Kreutz-Delgado K, Sivagnanam S, *et al.* (2021): The open EEGLAB portal Interface: High-Performance computing with EEGLAB. *NeuroImage* 224: 116778.

3. Pion-Tonachini L, Kreutz-Delgado K, Makeig S (2019): ICLabel: An automated electroencephalographic independent component classifier, dataset, and website. *Neuroimage* 198: 181–197.

4. Lachaux JP, Rodriguez E, Martinerie J, Varela FJ (1999): Measuring phase synchrony in brain signals. *Hum Brain Mapp* 8: 194–208.

5. Barkley RA (2011): *Barkley Adult ADHD Rating Scale-IV (BAARS-IV)*. Guilford Press.

6. Constantino JN, Gruber CP (2012): *Social Responsiveness Scale: SRS-2*. Western Psychological Services Torrance, CA.

7. Aydin Ü, Capp SJ, Tye C, Colvert E, Lau-Zhu A, Rijsdijk F, *et al.* (2022): Quality of life, functional impairment and continuous performance task event-related potentials (ERPs) in young adults with ADHD and autism: A twin study. *JCPP Advances* n/a: e12090.

8. Fayyad J, Graaf RD, Kessler R, Alonso J, Angermeyer M, Demyttenaere K, *et al.* (2007): Cross-national prevalence and correlates of adult attention-deficit hyperactivity disorder. *The British Journal of Psychiatry* 190: 402–409.

9. Colvert E, Tick B, McEwen F, Stewart C, Curran SR, Woodhouse E, *et al.* (2015): Heritability of Autism Spectrum Disorder in a UK Population-Based Twin Sample. *JAMA Psychiatry* 72: 415–423.

10. McLoughlin G, Palmer JA, Rijsdijk F, Makeig S (2014): Genetic Overlap between Evoked Frontocentral Theta-Band Phase Variability, Reaction Time Variability, and Attention-Deficit/Hyperactivity Disorder Symptoms in a Twin Study. *Biological Psychiatry* 75: 238–247.

11. Aydin Ü, Capp SJ, Tye C, Colvert E, Lau-Zhu A, Rijsdijk F, *et al.* (2022): Quality of life, functional impairment and continuous performance task event-related potentials (ERPs) in young adults with ADHD and autism: A twin study. *JCPP Advances* 2: e12090.
